# Efficacy of repellents against fleas and ticks: a field trial on free roaming dogs in Mali, West Africa

**DOI:** 10.3389/finsc.2026.1811490

**Published:** 2026-07-06

**Authors:** Amy Junnila, Edita E Revay, Mohamed Traore, Sekou F. Traore, Abdoul Habib Beavogui, Alexey M. Prozorov, Tatiana A. Prozorova, Julia S. Volkova, Aidas Saldaitis, Roman V. Yakovlev, Karen McKenzie, Rui-De Xue, Gunter C. Muller

**Affiliations:** 1 Malaria Research and Training Center, Faculty of Medicine, Pharmacy and Odonto-Stomatology, University of Sciences, Techniques and Technology of Bamako, Bamako, Mali; 2 Paleo-DNA Laboratory, Department of Anthropology, Lakehead University, Thunder Bay, ON, Canada; 3 Centre National de Formation et de Recherche en Santé Rurale de Maferinyah, Forécariah, Guinea; 4 Department of Medical Sciences, Gamal Abdel Nasser University, Conakry, Guinea; 5 Ludwig-Maximilians-University of Munich, Munich, Germany; 6 Institute of Molecular Biology and Biotechnology, Foundation for Research and Technology-Hellas, Heraklion, Greece; 7 Ulyanovsk State University, Ulyanovsk, Russia; 8 Nature Research Centre, Vilnius, Lithuania; 9 X-BIO Institute, University of Tyumen, Tyumen, Russia; 10 Institute of Biology, Ecology, Soil Science, Agriculture and Forestry, University of Tyumen, Tyumen, Russia; 11 McKenzie Consulting and Research, LLC, Melbourne, FL, United States; 12 Anastasia Mosquito Control District, St. Augustine, FL, United States

**Keywords:** dog collars, dog ectoparasites, *Eucalyptus citriodora* oil (H/C), field trial, flea and tick control, SpotOn repellent

## Abstract

**Introduction:**

This study evaluated the efficacy of repellent products in protecting free‑roaming dogs from flea and tick infestations in Mali, West Africa. The work included a comparative assessment of four commonly marketed collar‑type repelling devices—the ultrasonic collar (TICKLESS Mini Dog), the plastic botanical‑based collar (MEDIDOG Natürliches Zeckenhalsband), the ceramic‑bead collar with microbial coating (Bailey Bijoux), and the natural amber bead collar (PetLove Bernsteinkette)—alongside a biocidal SpotOn formulation (TGF X‑Line SpotOn) containing 9.9% *Eucalyptus citriodora* oil (H/C). These collar‑type products are widely promoted as non‑chemical alternatives but remain poorly supported by empirical evidence. Ultrasonic collars claim to emit high‑frequency sound waves intended to deter fleas and ticks, whereas ceramic, plastic, and amber collars rely on passive mechanisms such as surface charge, friction, or volatile compounds released from the materials. The SpotOn formulation, in contrast, functions through volatile plant compounds presumed to act on parasite chemoreceptors and reduce attachment without relying on insecticidal killing.

**Methods:**

The evaluation was conducted in two parallel phases. The first trial focused on small dogs (3–6 kg) to allow sensitive comparison across all repellent products. All repellents were tested together, with 10 dogs in the SpotOn group, 6 dogs assigned to each collar‑type treatment, and 10 untreated dogs as controls over an 86‑day study period. A second trial assessed the SpotOn formulation across a broader range of body sizes to determine whether efficacy was maintained when applied at the recommended dose for heavier dogs. In this phase, 40 dogs—20 medium (6–15 kg) and 20 large (15–30 kg)—were enrolled and monitored for reinfestation over the same period.

**Results:**

Laboratory assays confirmed that the SpotOn’s primary mode of action was repellency rather than insecticidal activity. Overall, the findings demonstrate that regular application of the SpotOn provides effective, long‑lasting protection against fleas and ticks under challenging field conditions, whereas none of the tested collar‑type products showed significant repellent effects.

## 1 Introduction

Fleas and ticks are among the most persistent and globally distributed ectoparasites affecting domestic dogs, posing substantial health risks to both animals and the humans who live alongside them. These arthropods are competent vectors for a wide range of pathogens, including *Ehrlichia canis*, *Babesia vogeli*, *B. canis*, and *Borrelia burgdorferi* (1–3). Their capacity to transmit pathogens, combined with their resilience across diverse climates, underscores the need for effective, sustainable, and accessible control strategies.

Among fleas, the cat flea (*Ctenocephalides felis*) is the most important species affecting companion animals worldwide. Originating in Africa (4), *C. felis* has become a highly successful invasive ectoparasite and is now the dominant flea species on both cats and dogs across Europe and West Africa. Although adult fleas are tightly host-dependent and survive only briefly off-host, they can persist for extended periods on suitable hosts and contribute to flea-bite dermatitis and transmission of *Dipylidium caninum*. Environmental development of immature stages and widespread insecticide resistance (5) further complicate management, making early prevention, particularly through repellents and trapping, an ecologically sound strategy.

Ticks present an equally significant challenge. The brown dog tick (*Rhipicephalus sanguineus*) is considered the most widespread tick species globally (6) and is now established across much of Europe, including regions previously considered climatically unsuitable (7). In Mali, it is the predominant tick infesting domestic dogs. Unusually, *R. sanguineus* can complete its entire life cycle both indoors and outdoors, enabling persistent infestations in kennels, homes, and other sheltered environments (8). It is a major vector of canine pathogens such as *Ehrlichia canis* and *Babesia canis*, and in some regions also transmits *Rickettsia conorii*, the agent of Mediterranean spotted fever (9). Preventing establishment in domestic environments remains the most effective strategy, and high-efficacy topical repellents continue to play a central role in reducing tick attachment and pathogen transmission.

Traditional ectoparasite control has relied heavily on synthetic insecticides and acaricides, but these approaches face increasing challenges, including resistance development, environmental concerns, and questions regarding long-term exposure in pets and humans (10, 11). Integrated management of ectoparasites of livestock as well as companion animals includes alternative strategies, such as the use of repellents in order to reduce the use of insecticides and/or acaricides to a minimum. Repellents are substances, which deter arthropods from flying to, landing on or biting humans or animals (12–14). In the EU, repellents are biocidal products with active ingredients, which need to be approved by the European Chemical Agency (ECHA). Repellents include synthetic substances and products that are based on natural active ingredients, which are often derived from essential oils. The most efficient and therefore widely used is *Eucalyptus citriodora* oil hydrated, cyclized (H/C), CAS 1245629-80-4. The raw material is derived from the leaves of lemon eucalyptus (*Corymbia citriodora*) by steam extraction and refined to the biocidal specific active substance *Eucalyptus citriodora* oil (H/C). It’s main component is para-menthane-3,8-diol (PMD), which has demonstrated repellent activity against a variety of hematophagous arthropods, including mosquitoes, fleas, and ticks (12, 13).

Mali provides a rigorous natural testing environment for evaluating such repellents due to its warm climate, high ectoparasite pressure, and year-round vector activity (15, 16). Free-roaming domestic dogs in rural communities experience continuous exposure to infested environments, unrestricted movement, and limited access to routine veterinary care, conditions that generate naturally high infestation rates and offer a realistic framework for assessing reinfestation dynamics (8, 17).

In this context, the study compared four commonly marketed collar-type repelling devices, ultrasonic, ceramic, plastic, and amber collars, which are widely promoted to pet owners despite lacking active substances with demonstrated biological activity against ectoparasites (18, 19). Ultrasonic collars are advertised as emitting high-frequency sound waves intended to deter fleas and ticks, whereas ceramic, plastic, and amber collars rely on passive mechanisms such as surface charge, friction, or volatile compounds released from the materials; none of these mechanisms have validated repellent or insecticidal effects (20–22). In contrast, the SpotOn formulation containing 9.9% *Eucalyptus citriodora* oil (H/C) functions through volatile plant-derived compounds that form a repellent barrier on the dog’s skin, reducing flea and tick attachment without relying on insecticidal killing (23, 24).

Conducted under demanding climatic conditions and high natural infestation pressure, this study contributes to the evaluation of alternative repellents for canine ectoparasite management. By focusing on free-roaming dogs in a real-world African setting, the research provides insights relevant to both tropical and temperate regions, where flea and tick activity is strongly seasonal. The findings highlight the need for integrated, sustainable approaches to ectoparasite control that combine effective repellents with broader strategies for animal health and vector management.

## 2 Materials and methods

### 2.1 Study area

The field trial was conducted in the Koulikoro Region of Mali, West Africa, across six rural villages located 60–80 kilometers southwest of Bamako. This heterogeneous landscape of forests, pastures, and agricultural fields creates consistently high ectoparasite pressure on domestic dogs. Warm temperatures, seasonal humidity, and the lack of any antiparasitic treatments of dogs, support stable populations of the cat flea (*Ctenocephalides felis*) and the brown dog tick (*Rhipicephalus sanguineus*), the two primary target species of this study.

Collaboration with local villagers and hunting associations facilitated recruitment of free-roaming domestic, hunting, and guard dogs. To minimize cross-contamination and ensure independence of observations, each household contributed only one dog. These free-roaming dogs experience continuous natural exposure to ectoparasites due to unrestricted movement and close interaction with human settlements, livestock, and wildlife.

### 2.2 Target organisms

The study area was selected based on prior evidence of substantial infestations with *C. felis* and *R. sanguineus*. Before enrollment, candidate dogs were screened through systematic combing and palpation to confirm active infestation and ensure inclusion of animals from areas with reliably high ectoparasite burdens.

Two days after screening, all fleas and ticks were removed and counted to establish a standardized baseline. Dogs were combed thoroughly, washed for five minutes with clear lukewarm water, and the wash-off was filtered through a fine sieve to recover remaining parasites. All recovered ectoparasites were identified and recorded, establishing the pre-treatment infestation level, and ensuring that dogs began the trial free of fleas and ticks so that subsequent reinfestation reflected natural exposure and treatment effects.

After washing, dogs were allowed to dry for 30 minutes before product application. This standardized wash-and-dry procedure was applied uniformly across all treatment groups. Although field staff were blinded to product identity for the SpotOn treatments through the use of a visually identical placebo formulation (sunflower oil), equivalent blinding was not possible for the collar products because each collar had a distinct appearance. As a result, observers were aware of which collar type each dog was wearing during data collection. This constitutes a potential source. To minimize the risk of observer bias, enumerators followed a standardized counting protocol and were not informed of the study hypotheses or expected product rankings.

### 2.3 Identification of the local flea and tick fauna

To document the ectoparasite community present at the study site, samples of fleas and ticks collected at the beginning and end of the trial were preserved in labeled vials for laboratory verification. Species identification was performed by experienced parasitologists at the Université des Sciences, des Techniques et des Technologies de Bamako (USTTB). This confirmed that the local ectoparasite fauna was dominated by *C. felis* and *R. sanguineus*, consistent with regional reports from West Africa. Accurate species identification ensured that trial outcomes could be interpreted in the context of the biology and vector potential of the local flea and tick populations.

### 2.4 Weather

The trial spanned from July 24, 2023, to April 17, 2024, covering both the rainy and early dry seasons. Weather data for 42 days of the study were obtained from the nearest station at USTTB (**Supplementary Figure 1**) but was only available from July 22nd to September 2nd. Although the available dataset does not cover the full annual cycle, it does capture the transition from peak rainy-season conditions (July–August) toward the onset of the dry season (September) in Bamako. This period illustrates the typical seasonal decline in rainfall and humidity that characterizes the shift from the wet to dry season in southern Mali. The rainy season was characterized by high temperatures, elevated humidity, and frequent showers, while the dry season showed declining rainfall and gradually rising temperatures.

### 2.5 Dog selection and grouping

When all repellent products were tested together (the four collar-type devices plus the SpotOn and its control), the study included 44 small dogs (3–6 kg). Of these, 20 dogs were assigned to the SpotOn and control groups, and 24 dogs were distributed across the collar-type treatments (N = 6 per collar type).

In the second parallel phase, which evaluated the SpotOn formulation for heavier dogs, 40 dogs were enrolled: 20 medium (6–15 kg) and 20 large (15–30 kg), half for treatment and half for control.

### 2.6 Test products, storage conditions, and trial period

All commercially available test products were received in Mali approximately three weeks before the start of the field trials. The four collar-type products included a ceramic-bead collar containing a proprietary microbial microbiome, a natural amber bead collar, an ultrasonic repellent device, and a plastic botanical-based collar containing plant-derived ingredients. The SpotOn formulation (9.9% *Eucalyptus citriodora* oil (H/C)) and a visually identical sunflower-oil placebo were also included. All field staff remained blinded to SpotOn product identity until data collection was complete.

The products were stored at room temperature in the USTTB analytical laboratory and applied strictly according to manufacturer or distributor instructions.

All test items were stored at room temperature in the USTTB analytical laboratory until deployment. The field trials commenced in mid-July 2023 and concluded in mid-April 2024, encompassing both dry and transitional climatic periods relevant to natural flea and tick activity in the study region.

### 2.7 Label claims

#### 2.7.1 X-Line SpotOn Fulltec (9.9% *Eucalyptus citriodora* oil (H/C))

The SpotOn product is marketed as a topical repellent for companion animals. According to the manufacturer, the formulation repels ticks and fleas on cats as well as on small, medium, and large dogs, providing immediate protection and helping to prevent new infestations for up to 12 weeks. For each weight category, small, medium and large, 3 pipet like cartridges with 2, 3 and 3ml are supplied in a box.

The product is described as spreading quickly across the skin to create a repellent barrier without stressing the treated animal, and it is suitable for use in young animals 12 weeks of age and older. In this study, different amounts of product are applied according to body-weight category (see below), and the label recommends reapplication every three weeks, during a 12-week period to maintain protection.

#### 2.7.2 TICKLESS^®^ ultrasonic device (ProtectONE Ltd.)

TICKLESS^®^ Mini Dog is an electronic device emitting ultrasound (>20 kHz), marketed to interfere with the orientation and host-finding behavior of ticks and fleas. Manufacturer claims include: prevention of worsening of existing infestations, reduction of infestations in some already-infested dogs, and prevention of new infestations in previously uninfested dogs. Effectiveness even in areas “heavily overspread with parasites”.

#### 2.7.3 MEDIDOG Natuerliches Zeckenhalsband (Bionic Nature GmbH & Co. KG)

The MEDIDOG Natuerliches Zeckenhalsband is a plastic collar marketed as being infused with a biocidal repellent containing 0.1% (1 g/kg) *Lavandula hybrida* extract, also known as lavandin oil (CAS 91722-69-9) as the active ingredient. Additionally, it contains essential oils derived from rockrose (Cistaceae).

According to the manufacturer, the collar begins to provide protection approximately one to two weeks after first use and remains effective for up to eight months. It is marketed as offering protection against ticks, fleas, and mites in both dogs and cats.

#### 2.7.4 PetLove amber necklace (Andras Kovacs)

The PetLove Amber Necklace consists of Baltic amber beads threaded onto a cord and is sold under the “Pest Control” category.

Although the product declaration does not make any explicit biocidal claims, the marketing of the product always refers to protection against tick infestation as a key promise.

#### 2.7.5 Effective microorganism ceramic collar (Bailey Bijoux)

**Figure 1:**
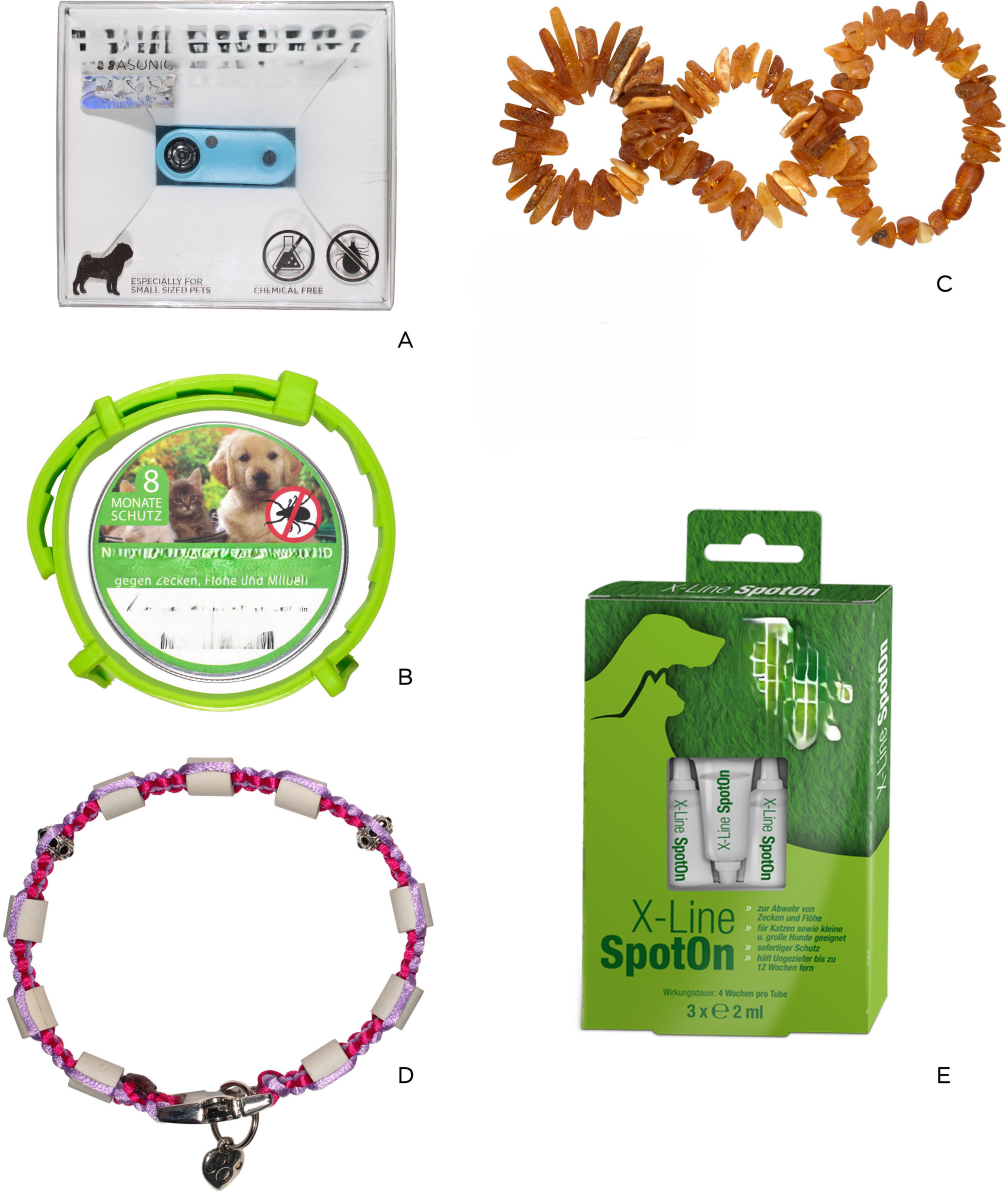
Test items evaluated during the field trial. **(A)** Ultrasonic collar (TICKLESS Mini Dog); **(B)** Plastic botanical-based collar (MEDIDOG Natuerliches Zeckenhalsband); **(C)** Ceramic-bead collar with microbial microbiome (Bailey Bijoux); **(D)** Natural amber bead collar (PetLove Bernsteinkette); **(E)** SpotOn formulation containing *Eucalyptus citriodora* oil (H/C) (9.9%) X-Line SpotOn.

The EM Ceramic Collar incorporates ceramic tubes containing a microbial consortium referred to as “Effective Microorganisms.” The manufacturer states that these ceramics emit electromagnetic resonance vibrations and infrared radiation, creating what is described as a regenerative and antioxidant environment. This environment is claimed to induce a form of “shock paralysis” in ticks, reducing their ability to attach or weakening their grip. Additional claims include reduced odor emission from the dog, improved immune function, increased vitality, enhanced overall well-being, and behavioral benefits such as calmer behavior in nervous dogs and greater stability in insecure dogs (see all test items in **Figure 1**).

### 2.8 Testing procedure

Dogs were first stratified by body-weight category (small, medium, large) and were then randomly assigned to treatment or control groups within each stratum. Baseline characteristics (initial infestation level, village, body weight, sex, age, and dog use) were comparable across groups.

The field evaluation was conducted in two phases. In the initial phase, all test items (the SpotOn formulation and the four collar-type repellent products) were tested exclusively on small dogs (3–6 kg). Each collar product was assessed using a cohort of 6 small dogs, while a 10 small-dog cohort was used to evaluate the SpotOn and a further 10 dogs for a placebo/control formulation. This design allowed direct comparison of all products under identical field conditions. When the preliminary assessments demonstrated that the collar-type products did not provide measurable repellency, subsequent testing focused solely on the SpotOn formulation.

In the second phase, the SpotOn was evaluated across two additional weight categories: medium (6–15 kg), and large (15–30 kg). Each weight cohort consisted of 20 free-roaming domestic dogs, with 10 dogs receiving the SpotOn formulation and 10 receiving the control formulation without active substance.

### 2.9 Standardized ectoparasite monitoring

Standardized ectoparasite counts were used to assess treatment efficacy. This method provides consistent, comparable measures of ectoparasite density before and after treatment without requiring quantification of the total parasite load on each animal. All dogs were evaluated using the same procedure by the same trained team throughout the study, ensuring methodological consistency and enabling robust statistical analysis.

Ectoparasite monitoring was performed by two experienced technicians, one examining the left side of each dog and the other the right. Using their fingers and a fine-toothed comb, technicians parted the coat in approximately 5-cm sections to expose the skin and detect fleas or ticks. Parasites were not removed during screening; instead, observations were recorded in real time by a supervising technician. Each dog was examined for five minutes, allowing complete coverage of the body surface. Counts from both technicians were pooled to generate a single ectoparasite value per dog per time point.

Monitoring was conducted in the late afternoon on Days 0, 1, 3, 5, 8, 12, 16, 20, 22, 26, 32, 38, 44, 50, 56, 62, 68, 74, 80, and 86. All collars were applied once at the start of the study, worn continuously, and not replaced during the trial. In contrast, the SpotOn formulation was reapplied at the manufacturer’s recommended interval of three weeks, with applications on Day 0, Day 20, and Day 44, and the monitoring schedule was structured to capture efficacy following each application.

### 2.10 Post-trial tick assessments

At the end of the field trial, remaining attached ticks on selected dogs in each weight class were treated with 0.5 mL of the SpotOn applied near the attachment site, and survival was assessed after 12 and 24 hours (**Supplementary Table 2**).

Additional assays exposed ticks and fleas to hair collected from treated and placebo-treated donor dogs. Hair was taken from the application zone, and parasite survival was assessed after 1 hour and 24 hours (**Supplementary Table 3**).

### 2.11 Application parameters

The test and control SpotOn formulations were supplied to the field team in coded, unlabeled containers to maintain blinding throughout the study. Because the original commercial packaging could not be used without revealing treatment identity, the products were transferred into identical containers and applied using standard 5 mL syringes. Application volumes were standardized by body-weight category: small dogs received 2 mL, medium dogs 3 mL, and large dogs 4 mL. The assigned volume was dispensed along the dorsal midline from the base of the head to the tail to ensure even distribution.

### 2.12 Dog weight measurement

Dog weights were recorded using medical-grade scales (Personenwaage KERN MPN 200K−1HM) equipped with a mother−child function. The dog owner’s weight was first tared to zero, after which the owner held the dog to obtain an accurate measurement. The scales provided readings with a precision of 100 g.

### 2.13 Data analysis and statistics

For each observation day, the average number of fleas or ticks per dog was calculated using the standard mean equation: (∑# of insects on dogs per day ÷ number of dogs).

The standard error (SE) was calculated by first determining the standard deviation (SD) and then applying: $SE=\frac{SD}{\surd N}$ where N represents the number of experimental repetitions.

Daily flea and tick counts were recorded for each dog throughout the study. To address the repeated-measures structure of the longitudinal dataset, the individual dog was treated as the experimental unit. When multiple products were compared, for each dog, the full time-series of parasite counts was integrated using the trapezoidal rule to generate an Area Under the Curve (AUC) value representing cumulative parasite burden over the observation period. This approach preserves the temporal pattern of infestation while avoiding pseudoreplication arising from treating repeated daily observations as independent.

AUC values for fleas and ticks were calculated separately for each dog and compared between treatment groups using a one-way ANOVA. For small dogs, where multiple products were evaluated, treatment effects were assessed using two-way ANOVA to compare the SpotOn formulation with the collar-type products. Effect sizes (η² and η²p) were calculated for the treatment factor and reported. Tukey’s *post hoc* test was used to make multiple comparisons between the products and the control. Statistical significance was defined as *P* < 0.05.

When comparing only the spot on to the control, the student’s t-test was used. All analyses were conducted using GraphPad Prism 9.00 for Windows (GraphPad Software, La Jolla, California, USA).

## 3 Results

### 3.1 Reinfestation testing on small dogs using all test items

Reinfestation monitoring on small dogs revealed a clear and consistent pattern across both fleas and ticks: the SpotOn was the only product that provided meaningful protection, while all four collar-type products performed no better than the untreated control.

#### 3.1.1 Fleas (all test items)

Following the post-wash baseline check, dogs experienced minor flea reinfestation up to Day 2. After this point, flea numbers on SpotOn-treated dogs dropped sharply and remained extremely low throughout the entire 86-day study period.

**Figure 2:**
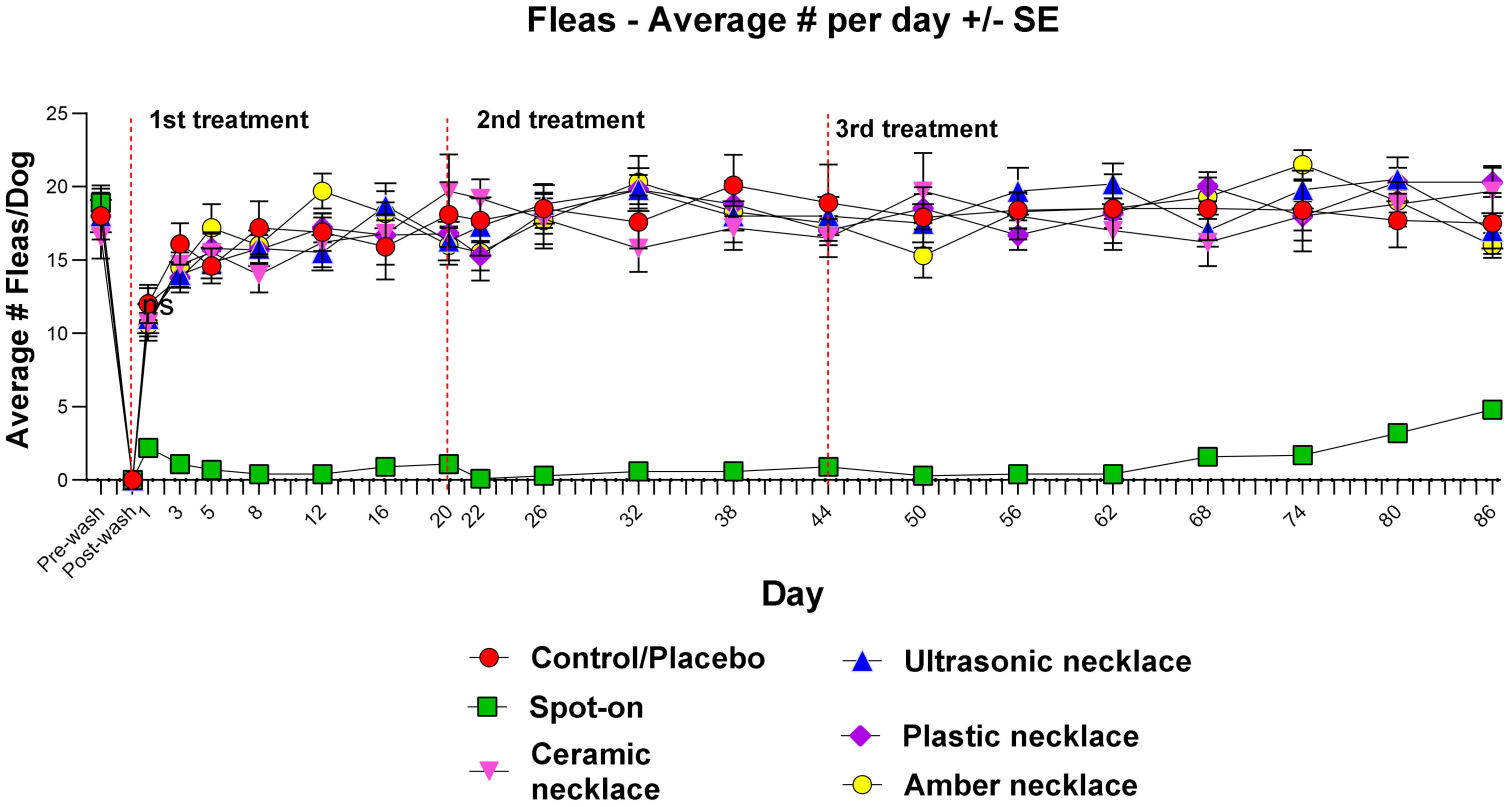
Longitudinal flea counts in treated and control dogs over an 86-day period.

The time-course data (**Figure 2**) show that the SpotOn maintained flea counts near zero after each application (Days 0, 20, and 44). The control group and all collar products (ultrasonic, plastic botanical, ceramic, and amber) showed nearly identical reinfestation curves, with consistently high flea numbers. No collar product demonstrated any detectable repellency effect.

Average number of fleas per small dog (± SE) recorded daily for six treatment groups: untreated control/placebo, SpotOn formulation, ultrasonic necklace, plastic necklace, ceramic necklace, and amber necklace. Vertical dashed lines indicate the timing of the first, second, and third treatments. Error bars represent the standard error of the mean for each observation day.

ANOVA comparisons confirmed these visual trends. SpotOn-treated dogs had dramatically lower mean flea counts than all other groups, with extremely strong statistical significance (P < 0.0001; **Table 1**). The effect size (η²) for the treatment factor in the full ANOVA was extremely large (η² = 0.75), indicating that 75% of the variance in landing counts was attributable to treatment group. Partial η² was also computed for the contrast between the SpotOn treatment and all collar products, which yielded similarly large values (average η²p is also 0.75 across post-wash timepoints). These effect sizes demonstrate that the study had more than sufficient power to detect even modest reductions in landing counts for collar products. The absence of significant effects for collars therefore reflects a true lack of efficacy rather than insufficient sample size.

**Table 1:** Tukey’s multiple comparisons (following one-way ANOVA) for average daily flea counts in small dogs across all treatments.

Test details	Mean 1	Mean 2	q	DF	P
SpotOn vs. Control/Placebo	1.933	16.6	15.66	120	P<0.0001
SpotOn vs. Ceramic collar	1.933	16.1	15.13	120	P<0.0001
SpotOn vs. Ultrasonic collar	1.933	16.56	15.62	120	P<0.0001
SpotOn vs. Plastic collar	1.933	16.53	15.59	120	P<0.0001
SpotOn vs. Amber collar	1.933	16.59	15.66	120	P<0.0001
Control/Placebo vs. Ceramic collar	16.6	16.1	0.53	120	P>0.9999
Control/Placebo vs. Ultrasonic collar	16.6	16.56	0.04	120	P>0.9999
Control/Placebo vs. Plastic collar	16.6	16.53	0.07	120	P>0.9999
Control/Placebo vs. Amber necklace	16.6	16.59	0.005	120	P>0.9999

Pairwise comparisons are shown for the SpotOn formulation and the untreated control versus each collar-type product (ceramic, ultrasonic, plastic, and amber). Values represent group means, Tukey’s *q* statistics, degrees of freedom (DF), and adjusted *P*-values for each contrast. Data reflect the average number of fleas per dog (± SE) recorded during the reinfestation period.

#### 3.1.2 Ticks (all test items)

A similar pattern was observed for ticks. After the initial post-wash period, small dogs experienced low-level reinfestation until Day 2. Beyond this point, the SpotOn kept tick numbers at consistently minimal levels, while all collar products tracked closely with the untreated control.

**Figure 3:**
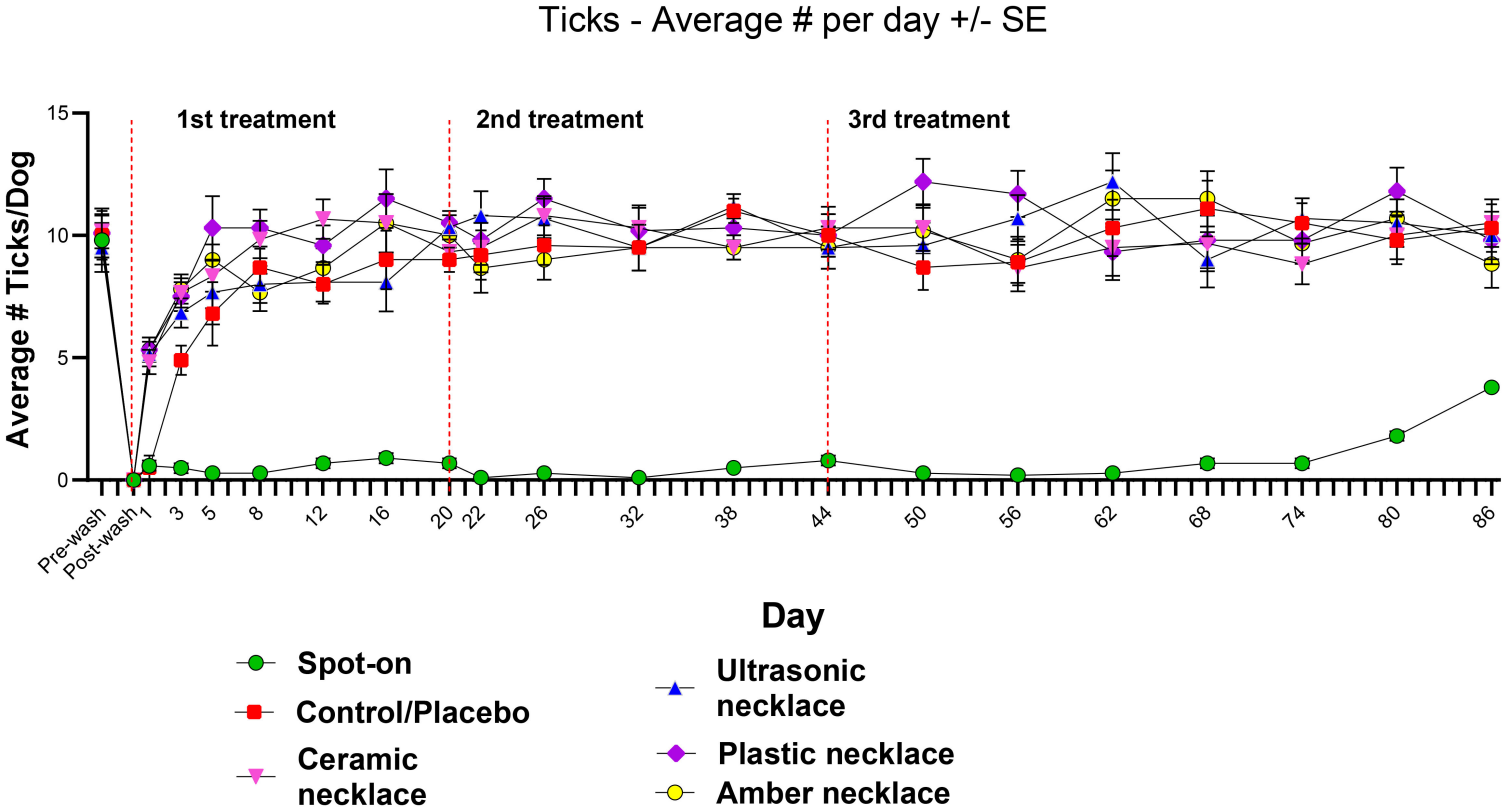
Longitudinal tick counts in treated and control dogs over an 86-day period.

The tick time-course data (**Figure 3**) show that the SpotOn maintained near-zero tick reinfestation after Day 2. The control and all collar products showed overlapping curves with high and persistent tick counts.

Average number of ticks per small dog (± SE) recorded daily for six treatment groups: untreated control/placebo, SpotOn formulation, ceramic necklace, ultrasonic necklace, plastic necklace, and amber necklace. Vertical dashed lines indicate the timing of the first, second, and third treatments. Error bars represent the standard error of the mean for each observation day.

None of the collar products produced any measurable reduction in tick reinfestation.

Statistical analysis again confirmed the superiority of the SpotOn. Across all comparisons, SpotOn vs. control, ceramic, ultrasonic, plastic, and amber, the differences were highly significant (P < 0.0001), with the same strong F-value pattern observed in the flea analysis (**Table 2**).

**Table 2:** Tukey’s multiple comparisons (following one-way ANOVA) for average daily tick counts in small dogs across all treatments.

Test details	Mean 1	Mean 2	q	DF	P
SpotOn vs. Control/Placebo	1.114	8.371	12.90	120	P<0.0001
SpotOn vs. Ceramic collar	1.114	9.009	14.03	120	P<0.0001
SpotOn vs. Ultrasonic collar	1.114	8.957	13.94	120	P<0.0001
SpotOn vs. Plastic collar	1.114	9.589	15.06	120	P<0.0001
SpotOn vs. Amber collar	1.114	8.887	13.82	120	P<0.0001
Control/Placebo vs. Ceramic collar	8.371	9.009	1.13	120	P>0.9999
Control/Placebo vs. Ultrasonic collar	8.371	8.957	1.04	120	P>0.9999
Control/Placebo vs. Plastic collar	8.371	9.589	2.16	120	P>0.9999
Control/Placebo vs. Amber collar	8.371	8.887	0.92	120	P>0.9999

Pairwise comparisons are shown for the SpotOn formulation and the untreated control versus each collar-type product (ceramic, ultrasonic, plastic, and amber). Values represent group means, Tukey’s *q* statistics, degrees of freedom (DF), and adjusted *P*-values for each contrast. Data reflect the average number of ticks per dog (± SE) recorded during the reinfestation period.

To evaluate whether sample size limited our ability to detect treatment effects, η² and partial η² were calculated for the treatment factor across all post-wash timepoints. The overall treatment effect was extremely large (average η² ≈ 0.57), indicating that 57% of the total variance in landing counts was attributable to treatment group. We also computed partial η² for the contrast between the SpotOn and each collar product, which yielded similarly large values (average η²p ≈ 0.58). These effect sizes demonstrate that the study had more than sufficient power to detect even modest differences between SpotOn and collar treatments.

### 3.2 Reinfestation testing on medium dogs using SpotOn

#### 3.2.1 Fleas (SpotOn – medium dogs)

**Figure 4:**
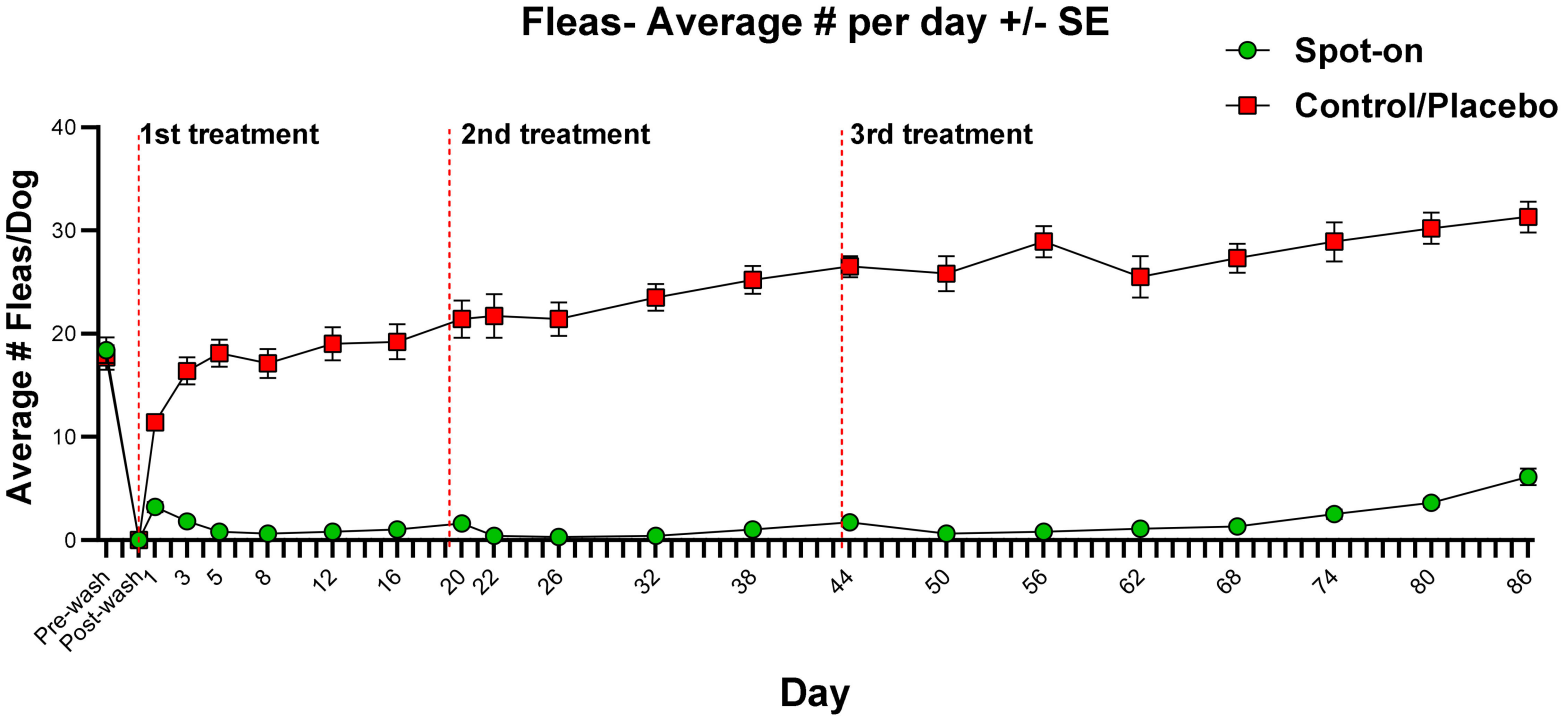
Longitudinal flea reinfestation in medium dogs following treatment with the SpotOn formulation. Average number of fleas per dog per day (± SE) recorded over the 63-day study period for medium-sized dogs treated with the test SpotOn formulation versus untreated controls. Vertical dashed lines indicate the timing of the first, second, and third treatments.

After the post-wash check, medium dogs showed low-level flea reinfestation up to Day 2, but these early infestations were rapidly suppressed by the test item. From Day 2 onward, flea numbers on treated dogs remained consistently and substantially lower than those observed in the control group. The time-course data (**Figure 4**) show a sharp decline in flea counts immediately after the first treatment, with flea numbers remaining low following each subsequent application, while control dogs maintained high and gradually increasing flea burdens throughout the study period.

Statistical analysis supported these findings. A paired t-test comparing the mean number of fleas on treated versus control medium dogs showed a highly significant difference, confirming the strong effect of the test item. Flea counts were consistently lower in the treated group across all 21 paired observations, and this reduction was statistically significant (t = 10.93, df = 20, P < 0.0001; two-tailed). These results reinforce the clear treatment-driven suppression of flea reinfestation in medium-sized dogs (**Table 3**).

**Table 3:** Paired t-test comparing average daily flea counts between treated and untreated medium dogs across the study period.

Medium dogs	
Paired t test	Fleas
P value	<0.0001
Significantly different (P < 0.05)?	Yes
One- or two-tailed P value?	Two-tailed
t, df	t=10.93, df=20
Number of pairs	21

Results summarize the paired analysis of mean flea counts for medium-sized dogs receiving the SpotOn formulation versus untreated controls. Values include the *t* statistic, degrees of freedom (df), two-tailed *P*-value, and the number of paired observations (n = 21).

#### 3.2.2 Ticks (SpotOn – medium dogs)

**Figure 5:**
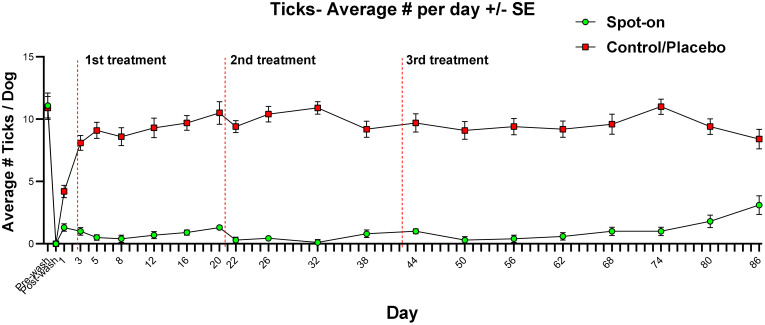
Longitudinal tick reinfestation in medium dogs following treatment with the SpotOn formulation. Average number of ticks per dog per day (± SE) recorded over the study period for medium-sized dogs treated with the test SpotOn formulation versus untreated controls. Vertical dashed lines indicate the timing of the first, second, and third treatments.

After the post-wash check, some low-level reinfestation of ticks occurred on treated medium dogs until Day 2, but these early infestations were quickly suppressed by the test item. From Day 2 onward, tick numbers on treated dogs remained consistently and markedly lower than those observed in the control group. The time-course data (**Figure 5**) show a sharp decline in tick counts immediately after the first treatment, with each subsequent application producing another rapid reduction, while control dogs maintained higher and relatively stable tick burdens throughout the study period.

Tick counts in medium dogs showed a highly significant reduction following treatment (**Table 4**). A paired t-test demonstrated a strong difference between treated and control conditions (t = 11.39, df = 20, two-tailed P < 0.0001). With 21 paired observations, the analysis confirmed that treatment effects were statistically significant at the P < 0.05 threshold.

**Table 4:** Paired t-test comparing average daily tick counts between treated and untreated medium dogs across the study period.

Medium dogs	
Paired t test	Ticks
P value	<0.0001
Significantly different (P < 0.05)?	Yes
One- or two-tailed P value?	Two-tailed
t, df	t=11.39, df=20
Number of pairs	21

Results summarize the paired analysis of mean tick counts for medium-sized dogs receiving the SpotOn formulation versus untreated controls. Values include the *t* statistic, degrees of freedom (df), two-tailed *P*-value, and the number of paired observations (n = 21).

### 3.3 Reinfestation testing on large dogs using SpotOn

#### 3.3.1 Fleas (spot-on – large dogs)

**Figure 6:**
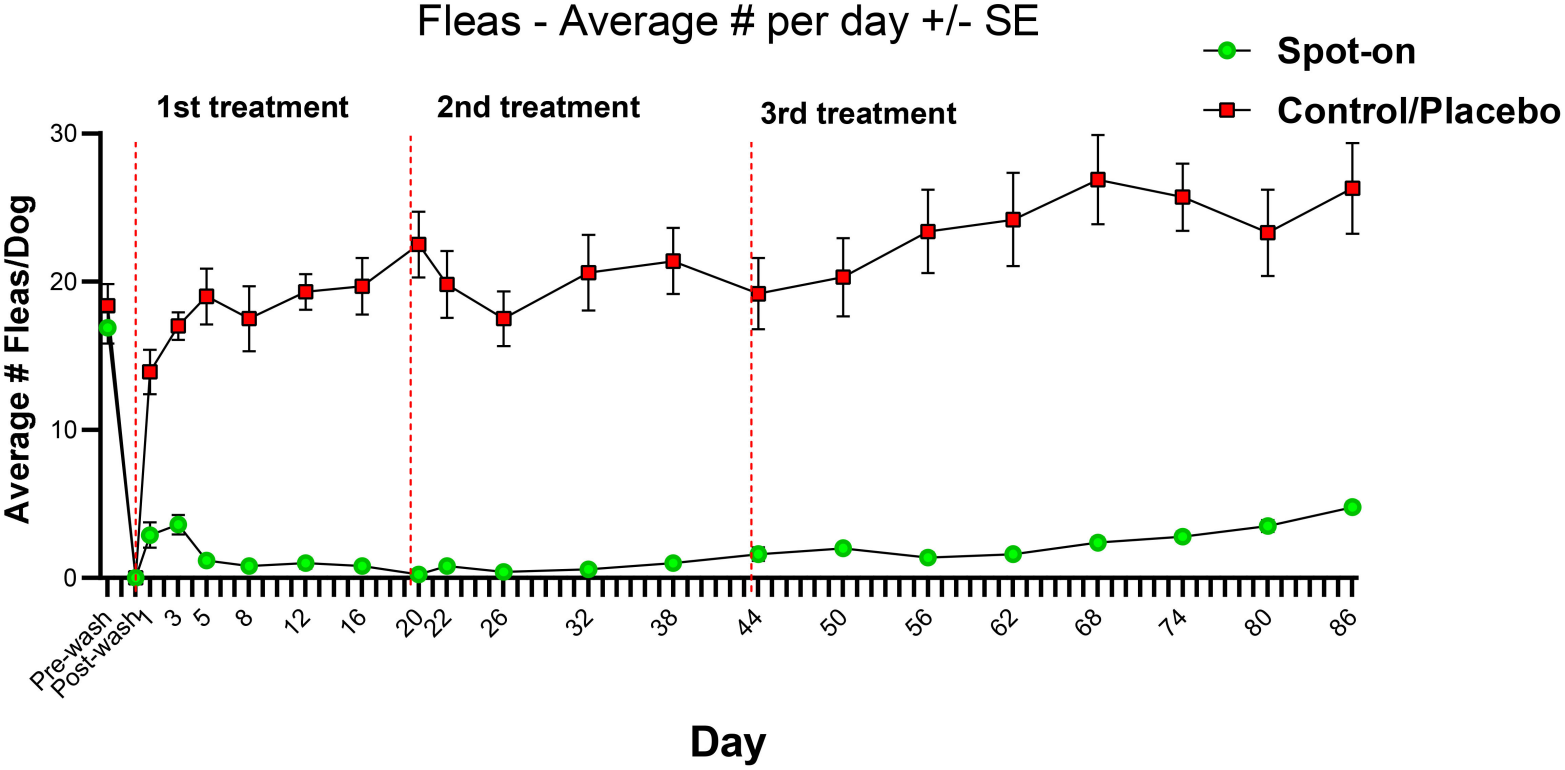
Longitudinal flea reinfestation in large dogs following treatment with the SpotOn formulation. Average number of fleas per dog per day (± SE) recorded over the study period for large-sized dogs treated with the test SpotOn formulation versus untreated controls. Vertical dashed lines indicate the timing of the first, second, and third treatments.

Mean flea counts for large dogs are shown in **Figure 6**. Before washing, both treated and control dogs carried approximately 20 fleas on average. Following cleaning at Day 0, all dogs were free of fleas. Immediately afterward, the untreated control group experienced a rapid and substantial increase in flea numbers, with counts rising sharply and remaining high throughout the study period. In contrast, treated dogs showed markedly lower reinfestation, with flea numbers dropping quickly after the first treatment and remaining low across subsequent observation days.

From Day 5 onward, flea counts on treated dogs continued to decline, demonstrating strong suppression of reinfestation pressure. A slight recovery of flea numbers was observed around Day 44, with more noticeable increases occurring near Day 74 when no further treatments were administered. Despite these late-period increases, flea burdens on treated dogs remained far below those of the control group for the duration of the study.

Statistical comparison of the full datasets confirmed a significant difference between treated and control groups (p < 0.0001; **Table 5**), demonstrating that the treatment produced a robust and sustained reduction in flea reinfestation in large dogs.

**Table 5:** Paired t-test comparing average daily flea counts between treated and untreated large dogs across the study period.

Large dogs	
Paired t test	Fleas
P value	<0.0001
Significantly different (P < 0.05)?	Yes
One- or two-tailed P value?	Two-tailed
t, df	t=12.53, df=20
Number of pairs	21

Results summarize the paired analysis of mean flea counts for large dogs receiving the SpotOn formulation versus untreated controls. Values include the *t* statistic, degrees of freedom (df), two-tailed *P*-value, and the number of paired observations (n = 21).

#### 3.3.2 Ticks (SpotOn – large dogs)

Tick counts differed sharply between treated and control dogs throughout the 86-day study period. Prior to treatment, both groups exhibited comparable levels of infestation. Following the first application of the SpotOn formulation on Day 0, tick numbers in treated dogs declined rapidly, reaching very low levels by approximately Day 5 and remaining consistently suppressed thereafter. In contrast, dogs in the placebo group showed a marked increase in tick burden shortly after the study began, with average counts rising to more than 10 ticks per dog during the early phase of the trial and remaining elevated throughout the study period.

**Figure 7:**
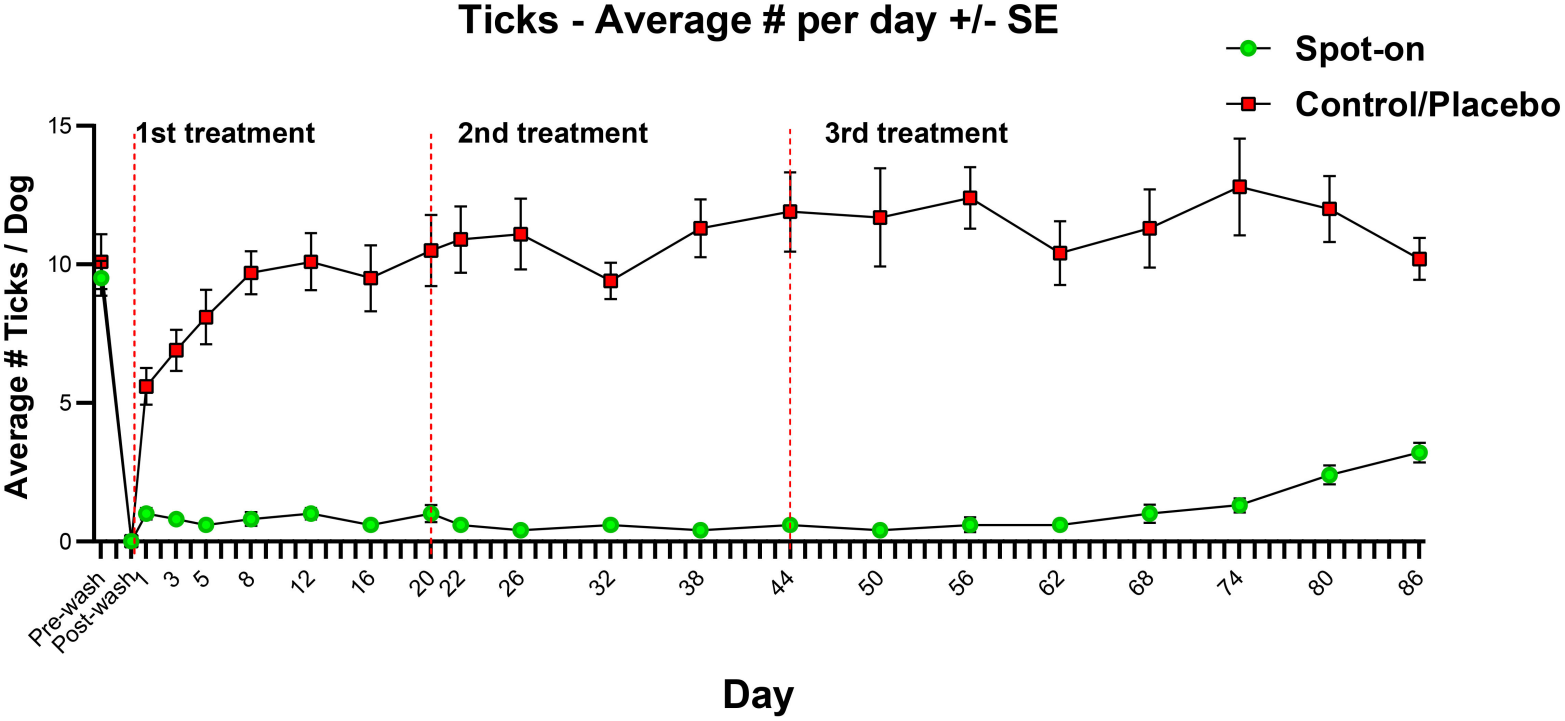
Longitudinal tick reinfestation in large dogs following treatment with the SpotOn formulation. Average number of ticks per dog per day (± SE) recorded over the study period for large-sized dogs treated with the test SpotOn formulation versus untreated controls. Vertical dashed lines indicate the timing of the first, second, and third treatments. Dogs receiving the SpotOn formulation maintained consistently low tick counts throughout the study, whereas control dogs exhibited persistently higher reinfestation levels.

The timing of the second and third SpotOn applications (Days 20 and 44) corresponded with continued maintenance of low tick counts in the treated group, with no evidence of breakthrough infestations even under sustained environmental pressure. Across the entire monitoring period, the SpotOn group maintained near-baseline infestation levels, while the control group experienced persistent and substantial reinfestation (**Figure 7**).

Across the full duration of the trial, the difference between treated and control groups was highly significant (p < 0.0001; **Table 6**), confirming that the treatment provided robust and long-lasting protection against tick reinfestation in large dogs.

**Table 6:** Paired t-test comparing average daily tick counts between treated and untreated large dogs across the study period.

Large dogs	
Paired t test	Ticks
P value	<0.0001
Significantly different (P < 0.05)?	Yes
One- or two-tailed P value?	Two-tailed
t, df	t=11.85, df=20
Number of pairs	21

Results summarize the paired analysis of mean tick counts for large dogs receiving the SpotOn formulation versus untreated controls. Values include the *t* statistic, degrees of freedom (df), two-tailed *P*-value, and the number of paired observations (n = 21).

## 4 Discussion

To our knowledge, this is the first published field study evaluating a SpotOn formulation containing the biocidal active *Eucalyptus citriodora* oil (H/C), CAS 1245629-80-4, for repelling fleas and ticks on dogs. Four collar-type products marketed with biocidal or repellent claims (ultrasonic, ceramic-bead, amber, and botanical-based collars) were also assessed for comparison.

The SpotOn formulation provided rapid and robust protection across all dog sizes, with flea and tick counts declining within 2 days of application. This onset aligns with the known diffusion dynamics of topical repellents, in which volatile compounds spread over the skin via the carrier formulation (14). Repellency remained high from Day 3 through Day 74, supported by reapplications on Days 20 and 44, and is consistent with the manufacturer’s 12-week protection claim and established ectoparasiticide evaluation guidelines (18). These findings confirm that each application provides at least 21 days of effective protection.

The active substance*, Eucalyptus citriodora* oil (H/C), contains volatile monoterpenoids that interfere with arthropod host-seeking behavior rather than exerting toxic effects (14). This repellent mode of action is well documented across multiple vector species (12, 13). Laboratory assays using hair clippings from treated dogs demonstrated nearly 100% survival for both fleas and ticks over 24 hours, confirming the absence of insecticidal activity. The low level of lethality, combined with the natural molecular diversity of the oil, suggests a low likelihood of resistance development. Treated dogs showed no stress responses, supporting the product’s suitability for routine use.

In contrast, none of the collar-type products—TICKLESS^®^, the Natural Tick Collar, the PetLove Amber Necklace, or the EM Ceramic Collar—showed measurable protective efficacy at any time point. Their advertised mechanisms (ultrasonic disruption, botanical vapor release, or “electromagnetic resonance”) lack empirical support, and previous studies similarly report poor performance of passive repellent devices under natural exposure conditions (12, 13). The present findings reinforce this evidence base, demonstrating that these collars do not provide meaningful protection against fleas or ticks.

The trial was conducted under severe environmental pressure, including high temperatures, high humidity, and peak seasonal ectoparasite abundance—conditions known to intensify flea and tick challenges (8, 15). Free-roaming hunting and guard dogs were continuously exposed to *Ctenocephalides felis* and *Rhipicephalus sanguineus*, species with comparable ecological behavior in West Africa, Europe, and North America (1, 2). Despite these demanding conditions, the SpotOn maintained strong protection and significantly reduced reinfestation, demonstrating robust field performance.

This is particularly relevant given rising insecticide and acaricide resistance (10, 11) and the increasing need for effective, non-insecticidal alternatives for integrated ectoparasite management.

Although spot-on treatments were fully blinded through the use of a placebo formulation, the collar products could not be blinded because each collar type had a distinct physical appearance. As a result, observers were aware of the collar assignment during ectoparasite monitoring. This constitutes a potential source of observer bias; however, all enumerators followed a standardized, hypothesis-neutral protocol focused solely on counting landings and bites, and they were not informed of expected product performance. While subtle observer effects cannot be entirely excluded, the objective nature of the primary endpoints and the consistency of patterns across treatment groups suggest that any influence of partial unblinding on the results was minimal.

Overall, the results demonstrate that the SpotOn formulation provides reliable, long-lasting, and environmentally resilient protection against fleas and ticks in dogs, under the conditions used in this study. Its rapid onset, strong repellent action, and sustained efficacy under high-challenge conditions support its use as a practical, scientifically grounded tool for reducing ectoparasite burden and associated disease risks across diverse geographic settings.

## Data Availability

The raw data supporting the conclusions of this article will be made available by the authors, without undue reservation.

## Supplementary material

Supplementary Figure 1 Weather data collected from July 22^nd^ to September 2^nd^ at the local USTTB weather monitoring station in Bamako.

Supplementary Table 2 Ticks falling from dogs in individual tents were collected and placed in Petri dishes for 24 hrs to assess survival. The number of surviving ticks was recorded after 12 hours and 24 hours.

Supplementary Table 3 The survival of fleas and ticks exposed directly to the test item in a Petri dish for 30 minutes, 1 hour and 24 hours was observed.
