# Supplementary material for: Efficacy of repellents against fleas and ticks: a field trial on free roaming dogs in Mali, West Africa
Source: Front Insect Sci. 2026 Jul 6;6:1811490. doi: 10.3389/finsc.2026.1811490 (PMC13382635; doi:10.3389/finsc.2026.1811490)

Figure S1: Weather data collected from July 22nd to September 2^nd^ at the local USTTB weather monitoring station in Bamako.


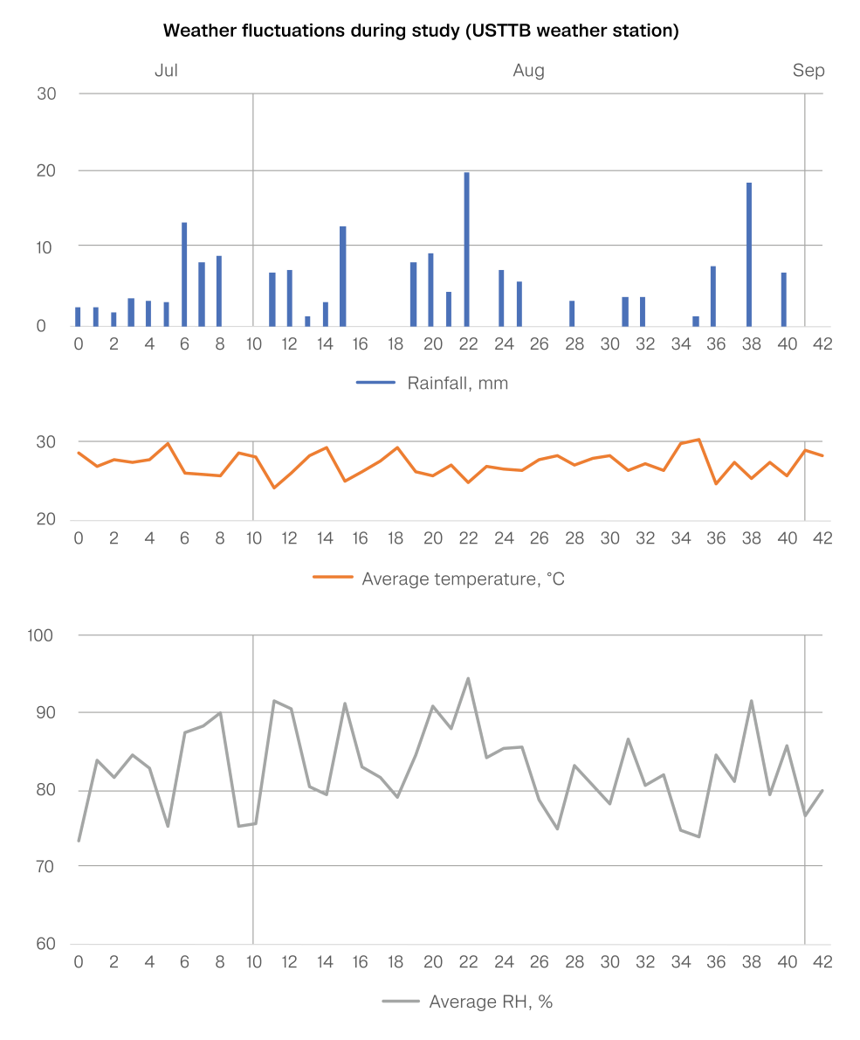


Table S1: Ticks falling from dogs in individual tents were collected and placed in Petrie dishes for 24 hrs to assess survival. The number of surviving ticks was recorded after 12 hours and 24 hours.


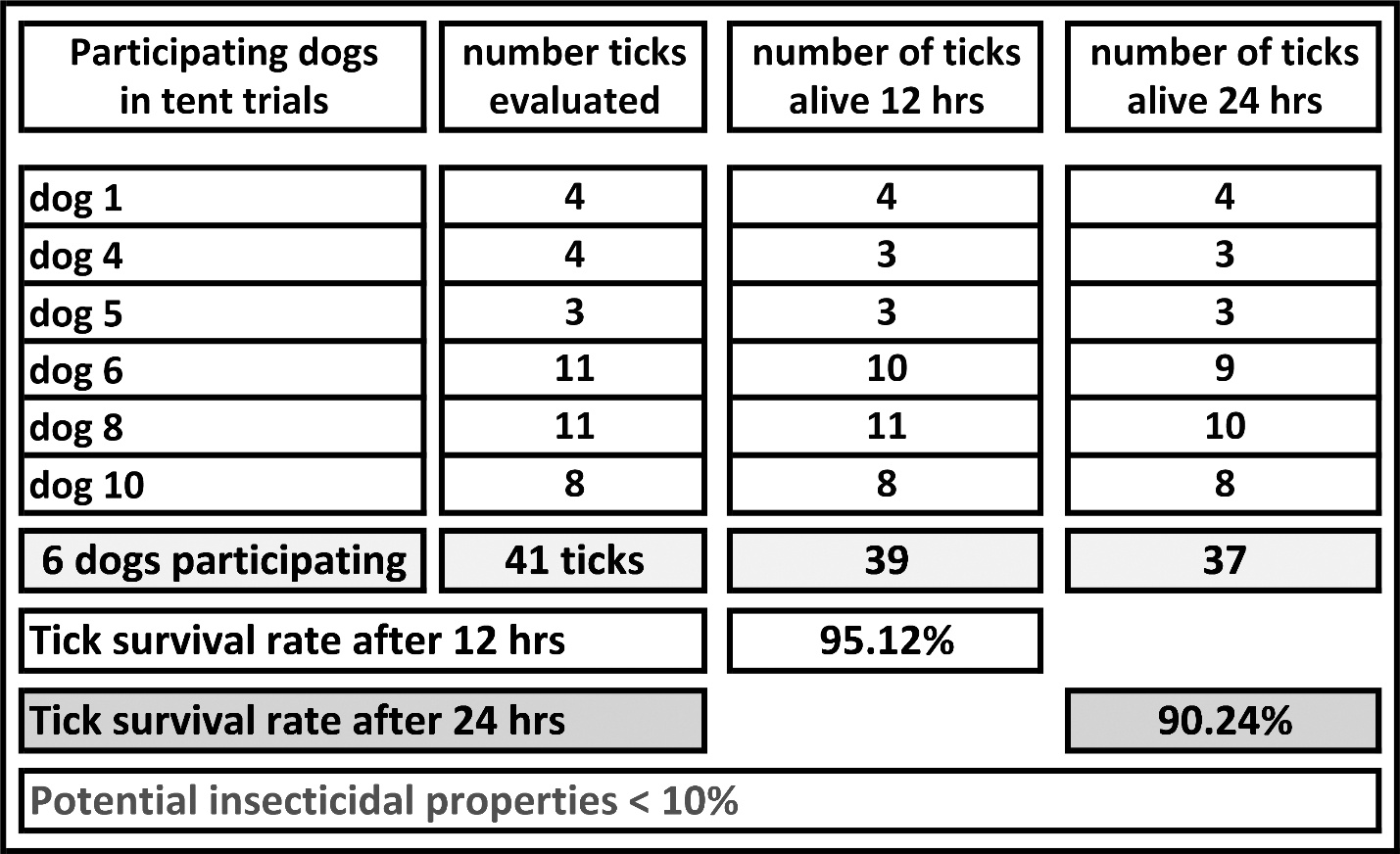


Table S2: The survival of fleas and ticks exposed directly to the test item in a Petri dish for 30 minutes, 1 hour and 24 hours, was observed.


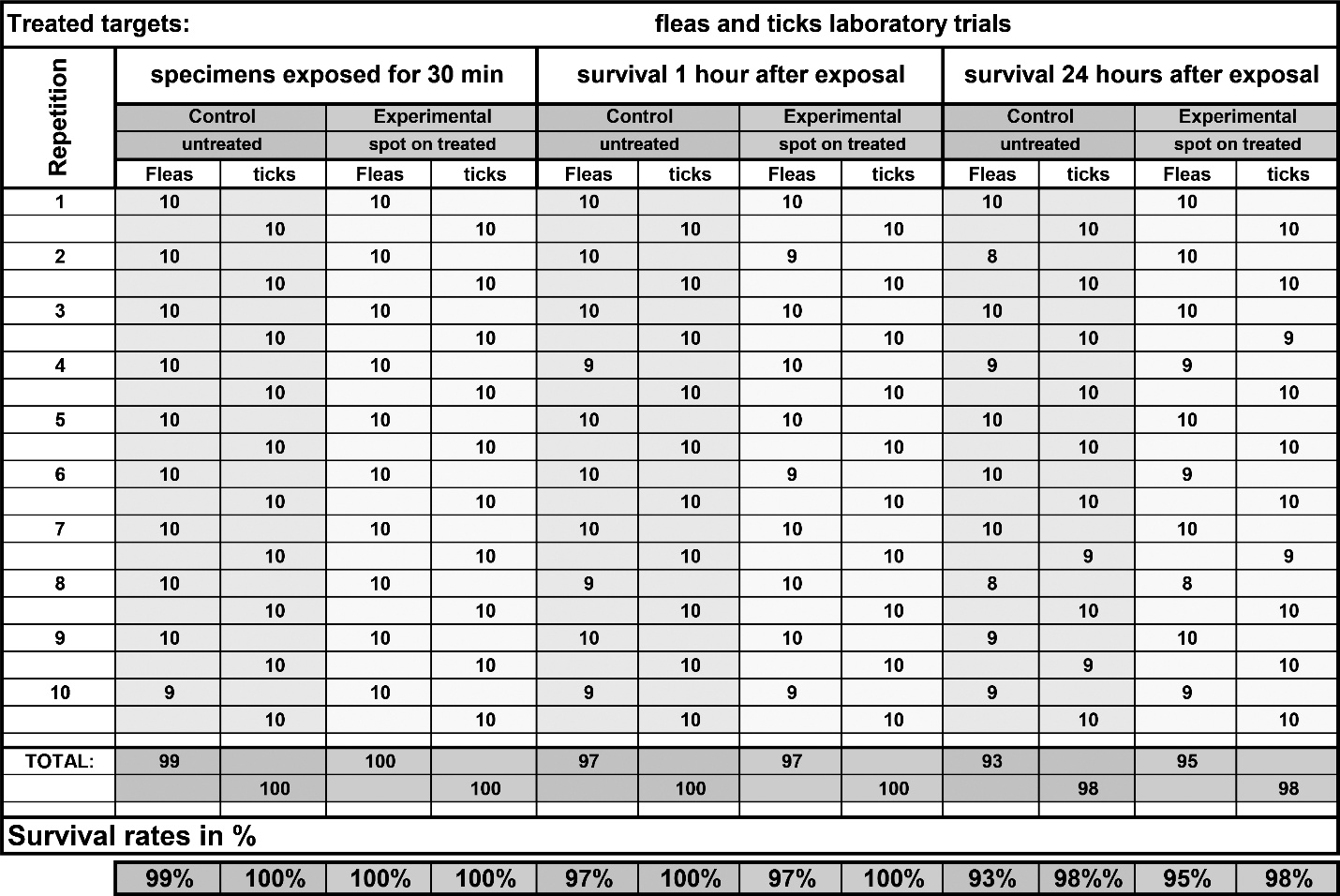

Supplement: Supplementary Figure 1 — Weather data collected from July 22nd to September 2nd at the local USTTB weather monitoring station in Bamako. [file SupplementaryFile1.docx]
